# RNA PROCESSING MECHANISMS DURING AGING AND CALORIC RESTRICTION

**DOI:** 10.1093/geroni/igac059.073

**Published:** 2022-12-20

**Authors:** Timothy Rhoads, Josef Clark, Rozalyn Anderson

**Affiliations:** University of Wisconsin-Madison, Madison, Wisconsin, United States; University of Wisconsin-Madison, Madison, Wisconsin, United States; University of Wisconsin-Madison, Madison, Wisconsin, United States

## Abstract

Caloric restriction (CR), a reduction in caloric intake without malnutrition, was first described in the 1930s and is a robust model to extend lifespan. While the specific mechanisms that produce delayed aging are still unknown, work has demonstrated the importance of metabolism – dysregulated mitochondrial metabolism is an aging hallmark, while CR upregulates energy metabolism. Crucially, transcriptional regulation plays a key role in implementing this metabolic program although details are still unclear. Splicing factors that are required for the beneficial effects of dietary restriction in nematodes have been identified, and large-scale exon usage changes have been observed across the metabolic network engaged by CR in various tissues from non-human primates, suggesting a translational mechanism that might contribute to how CR delays aging. We will discuss the landscape of RNA processing across multiple organism, tissue, and intervention contexts and provide a broad perspective of how RNA processing contributes to enhanced longevity.

